# Isopropyl 4-nitro­benzoate

**DOI:** 10.1107/S1600536811039407

**Published:** 2011-09-30

**Authors:** Pei Zou, Min-Hao Xie, Hao Wu, Ya-Ling Liu, Zheng-Ping Chen

**Affiliations:** a Jiangsu Institute of Nuclear Medicine, Wuxi 214063, People’s Republic of China

## Abstract

In the mol­ecule of the title compound, C_10_H_11_NO_4_, the nitro group is approximately coplanar with the benzene ring [dihedral angle = 4.57 (10)°], while the carboxyl­ate group is slightly twisted, making an angle of 12.16 (8)°. In the crystal, weak inter­molecular C—H⋯O hydrogen bonding and π–π stacking inter­actions [centroid–centroid distances = 3.670 (2) and 3.665 (2) Å] are observed.

## Related literature

For applications of benzoates in the chemistry of pigments and pharmaceuticals, see: Zhang *et al.* (1990 ▶, 1995 ▶). For a related structure, see: Wu *et al.* (2009 ▶).
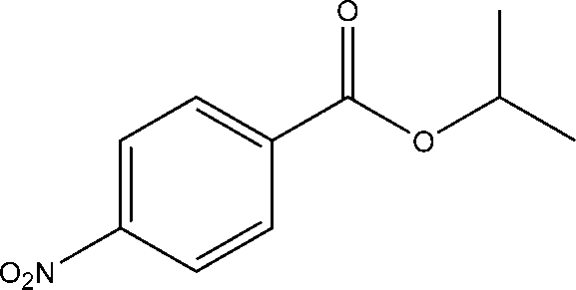


## Experimental

### 

#### Crystal data

C_10_H_11_NO_4_
*M* *_r_* = 209.20
Triclinic, 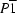

*a* = 6.729 (4) Å
*b* = 7.192 (4) Å
*c* = 10.388 (6) Å
α = 94.751 (9)°
β = 92.503 (7)°
γ = 95.901 (10)°
*V* = 497.6 (5) Å^3^
*Z* = 2
Mo *K*α radiation
μ = 0.11 mm^−1^
*T* = 153 K
0.37 × 0.33 × 0.10 mm

#### Data collection

Rigaku SPIDER diffractometer
6626 measured reflections
2862 independent reflections
1947 reflections with *I* > 2σ(*I*)
*R*_int_ = 0.023

#### Refinement

*R*[*F* ^2^ > 2σ(*F* ^2^)] = 0.046
*wR*(*F* ^2^) = 0.150
*S* = 1.00
2862 reflections
138 parameters
H-atom parameters constrained
Δρ_max_ = 0.34 e Å^−3^
Δρ_min_ = −0.29 e Å^−3^

### 

Data collection: *RAPID-AUTO* (Rigaku, 2004 ▶); cell refinement: *RAPID-AUTO*; data reduction: *RAPID-AUTO*; program(s) used to solve structure: *SHELXS97* (Sheldrick, 2008 ▶); program(s) used to refine structure: *SHELXL97* (Sheldrick, 2008 ▶); molecular graphics: *SHELXTL* (Sheldrick, 2008 ▶); software used to prepare material for publication: *SHELXTL*.

## Supplementary Material

Crystal structure: contains datablock(s) I, global. DOI: 10.1107/S1600536811039407/xu5329sup1.cif

Structure factors: contains datablock(s) I. DOI: 10.1107/S1600536811039407/xu5329Isup2.hkl

Supplementary material file. DOI: 10.1107/S1600536811039407/xu5329Isup3.cml

Additional supplementary materials:  crystallographic information; 3D view; checkCIF report

## Figures and Tables

**Table 1:** Hydrogen-bond geometry (Å, °)

*D*—H⋯*A*	*D*—H	H⋯*A*	*D*⋯*A*	*D*—H⋯*A*
C1—H1⋯O4^i^	0.95	2.46	3.311 (3)	149
C4—H4⋯O2^ii^	0.95	2.46	3.294 (3)	147

Symmetry codes: (i) 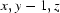; (ii) 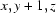.

**Fig. 1.**
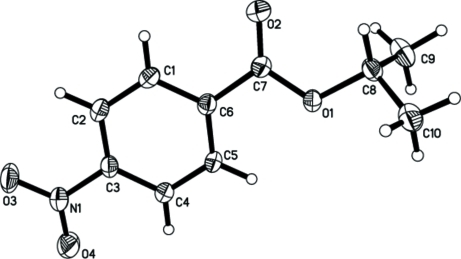
A view of the title compound with the atomic numbering scheme. Displacement ellipsoids were drawn at the 50% probability level.

## supplementary crystallographic information

### Comment

Benzoates are important intermediates in the chemistry of pigments and pharmaceuticals, which are widely used all over the world (Zhang *et al.*, 1995; Zhang *et al.*, 1990). The crystal structure of methyl 4-nitrobenzoate has been reported (Wu *et al.*, 2009). As an extension of our study, we report here the crystal structure of the title compound.

In the structure of the title compound (Fig. 1) the bond lengths and angles are within expected ranges. The nitro substituent group is nearly coplanar with the benzene ring (dihedral angle, 4.57 (10)°), while the ester group forms a dihedral angle of 12.16 (8)° with the benzene ring. In the crystal structure, adjacent molecules are linked together by weak C—H···O hydrogen bonds (Table 1). π-π stacking is observed between parallel benzene rings, centroids distances being 3.670 (2) [symmetry code -x,1-y,1-z] and 3.665 (2) Å [symmetry code 1-x,1-y,1-z].

### Experimental

A sample of commercial isopropyl 4-nitrobenzoate was crystallized by slow evaporation of a solution in methanol, colorless platelet-shaped crystals were formed after several days.

### Refinement

Positional parameters of all the H atoms bonds to C atoms were calculated geometrically and were allowed to ride on the C atoms to which they are bonded, with C_aromatic_—H = 0.95 Å, *U*_iso_(H) = 1.2*U*_eq_(C_aromatic_); C_methyl_—H = 0.98 Å, *U*_iso_(H) = 1.5*U*_eq_(C_methyl_) and C_methylidyne_—H = 1.00 Å, *U*_iso_(H) = 1.2*U*_eq_(C_methylidyne_).

### Crystal data

**Table:**

C_10_H_11_NO_4_	*Z* = 2
*M**_r_* = 209.20	*F*(000) = 220
Triclinic, *P*1	*D*_x_ = 1.396 Mg m^−^^3^
Hall symbol: -P 1	Mo *K*α radiation, λ = 0.71073 Å
*a* = 6.729 (4) Å	Cell parameters from 1490 reflections
*b* = 7.192 (4) Å	θ = 2.9–30.0°
*c* = 10.388 (6) Å	µ = 0.11 mm^−^^1^
α = 94.751 (9)°	*T* = 153 K
β = 92.503 (7)°	Platelet, colorless
γ = 95.901 (10)°	0.37 × 0.33 × 0.10 mm
*V* = 497.6 (5) Å^3^	

### Data collection

**Table:**

Rigaku SPIDER diffractometer	1947 reflections with *I* > 2σ(*I*)
Radiation source: Rotating Anode	*R*_int_ = 0.023
graphite	θ_max_ = 30.0°, θ_min_ = 2.0°
ω scans	*h* = −9→9
6626 measured reflections	*k* = −10→9
2862 independent reflections	*l* = −14→14

### Refinement

**Table:**

Refinement on *F*^2^	Primary atom site location: structure-invariant direct methods
Least-squares matrix: full	Secondary atom site location: difference Fourier map
*R*[*F*^2^ > 2σ(*F*^2^)] = 0.046	Hydrogen site location: inferred from neighbouring sites
*wR*(*F*^2^) = 0.150	H-atom parameters constrained
*S* = 1.00	*w* = 1/[σ^2^(*F*_o_^2^) + (0.071*P*)^2^ + 0.196*P*] where *P* = (*F*_o_^2^ + 2*F*_c_^2^)/3
2862 reflections	(Δ/σ)_max_ < 0.001
138 parameters	Δρ_max_ = 0.34 e Å^−^^3^
0 restraints	Δρ_min_ = −0.29 e Å^−^^3^

### Special details

**Table:**

Geometry. All e.s.d.'s (except the e.s.d. in the dihedral angle between two l.s. planes) are estimated using the full covariance matrix. The cell e.s.d.'s are taken into account individually in the estimation of e.s.d.'s in distances, angles and torsion angles; correlations between e.s.d.'s in cell parameters are only used when they are defined by crystal symmetry. An approximate (isotropic) treatment of cell e.s.d.'s is used for estimating e.s.d.'s involving l.s. planes.
Refinement. Refinement of *F*^2^ against ALL reflections. The weighted *R*-factor *wR* and goodness of fit *S* are based on *F*^2^, conventional *R*-factors *R* are based on *F*, with *F* set to zero for negative *F*^2^. The threshold expression of *F*^2^ > σ(*F*^2^) is used only for calculating *R*-factors(gt) *etc*. and is not relevant to the choice of reflections for refinement. *R*-factors based on *F*^2^ are statistically about twice as large as those based on *F*, and *R*- factors based on ALL data will be even larger.

### Fractional atomic coordinates and isotropic or equivalent isotropic displacement parameters (Å^2^)

**Table:**

	*x*	*y*	*z*	*U*_iso_*/*U*_eq_	
O1	0.24504 (18)	0.35230 (15)	0.12880 (10)	0.0258 (3)	
O2	0.3016 (2)	0.12996 (17)	0.26213 (12)	0.0375 (3)	
O3	0.2023 (2)	0.79014 (18)	0.77175 (11)	0.0381 (3)	
O4	0.2382 (3)	1.00939 (19)	0.64283 (13)	0.0513 (4)	
N1	0.2278 (2)	0.8444 (2)	0.66434 (13)	0.0288 (3)	
C1	0.2535 (2)	0.3845 (2)	0.47750 (14)	0.0226 (3)	
H1	0.2540	0.2559	0.4922	0.027*	
C2	0.2421 (2)	0.5176 (2)	0.58075 (14)	0.0233 (3)	
H2	0.2325	0.4818	0.6665	0.028*	
C3	0.2450 (2)	0.7032 (2)	0.55569 (14)	0.0221 (3)	
C4	0.2586 (2)	0.7627 (2)	0.43241 (14)	0.0234 (3)	
H4	0.2618	0.8919	0.4186	0.028*	
C5	0.2672 (2)	0.6275 (2)	0.32976 (14)	0.0223 (3)	
H5	0.2752	0.6638	0.2441	0.027*	
C6	0.2642 (2)	0.4393 (2)	0.35198 (14)	0.0207 (3)	
C7	0.2732 (2)	0.2890 (2)	0.24432 (14)	0.0225 (3)	
C8	0.2391 (2)	0.2167 (2)	0.01385 (15)	0.0252 (3)	
H8	0.2027	0.0870	0.0392	0.030*	
C9	0.4432 (3)	0.2293 (3)	−0.04028 (18)	0.0374 (4)	
H9A	0.4766	0.3551	−0.0682	0.056*	
H9B	0.4435	0.1358	−0.1145	0.056*	
H9C	0.5424	0.2052	0.0265	0.056*	
C10	0.0769 (3)	0.2689 (3)	−0.07652 (17)	0.0376 (4)	
H10A	−0.0497	0.2638	−0.0331	0.056*	
H10B	0.0624	0.1806	−0.1543	0.056*	
H10C	0.1125	0.3963	−0.1010	0.056*	

### Atomic displacement parameters (Å^2^)

**Table:**

	*U*^11^	*U*^22^	*U*^33^	*U*^12^	*U*^13^	*U*^23^
O1	0.0401 (7)	0.0214 (5)	0.0164 (5)	0.0043 (4)	0.0039 (4)	0.0014 (4)
O2	0.0614 (9)	0.0256 (6)	0.0276 (6)	0.0129 (6)	0.0017 (6)	0.0047 (5)
O3	0.0533 (8)	0.0423 (7)	0.0184 (6)	0.0022 (6)	0.0076 (5)	0.0014 (5)
O4	0.0989 (13)	0.0302 (7)	0.0272 (7)	0.0169 (7)	0.0103 (7)	0.0003 (5)
N1	0.0359 (8)	0.0324 (7)	0.0185 (6)	0.0060 (6)	0.0028 (5)	0.0004 (5)
C1	0.0229 (7)	0.0240 (7)	0.0218 (7)	0.0027 (6)	0.0012 (5)	0.0071 (5)
C2	0.0234 (7)	0.0296 (8)	0.0174 (6)	0.0024 (6)	0.0014 (5)	0.0061 (5)
C3	0.0223 (7)	0.0275 (7)	0.0165 (6)	0.0035 (6)	0.0021 (5)	0.0006 (5)
C4	0.0290 (8)	0.0229 (7)	0.0191 (7)	0.0046 (6)	0.0025 (6)	0.0035 (5)
C5	0.0265 (8)	0.0257 (7)	0.0158 (6)	0.0043 (6)	0.0036 (5)	0.0047 (5)
C6	0.0197 (7)	0.0245 (7)	0.0183 (6)	0.0030 (5)	0.0021 (5)	0.0034 (5)
C7	0.0245 (7)	0.0234 (7)	0.0201 (7)	0.0030 (6)	0.0030 (5)	0.0041 (5)
C8	0.0341 (9)	0.0198 (7)	0.0209 (7)	0.0016 (6)	0.0039 (6)	−0.0021 (5)
C9	0.0406 (10)	0.0350 (9)	0.0352 (9)	0.0023 (7)	0.0103 (8)	−0.0087 (7)
C10	0.0462 (11)	0.0389 (10)	0.0276 (8)	0.0134 (8)	−0.0034 (7)	−0.0064 (7)

### Geometric parameters (Å, °)

**Table:**

O1—C7	1.3309 (18)	C4—H4	0.9500
O1—C8	1.4741 (18)	C5—C6	1.390 (2)
O2—C7	1.2067 (19)	C5—H5	0.9500
O3—N1	1.2255 (18)	C6—C7	1.497 (2)
O4—N1	1.221 (2)	C8—C9	1.503 (2)
N1—C3	1.471 (2)	C8—C10	1.506 (2)
C1—C2	1.387 (2)	C8—H8	1.0000
C1—C6	1.396 (2)	C9—H9A	0.9800
C1—H1	0.9500	C9—H9B	0.9800
C2—C3	1.380 (2)	C9—H9C	0.9800
C2—H2	0.9500	C10—H10A	0.9800
C3—C4	1.387 (2)	C10—H10B	0.9800
C4—C5	1.390 (2)	C10—H10C	0.9800
			
C7—O1—C8	117.82 (12)	O2—C7—O1	124.91 (14)
O4—N1—O3	123.35 (14)	O2—C7—C6	123.18 (14)
O4—N1—C3	118.42 (13)	O1—C7—C6	111.91 (13)
O3—N1—C3	118.23 (14)	O1—C8—C9	108.43 (13)
C2—C1—C6	120.08 (14)	O1—C8—C10	105.50 (13)
C2—C1—H1	120.0	C9—C8—C10	114.14 (15)
C6—C1—H1	120.0	O1—C8—H8	109.5
C3—C2—C1	118.24 (14)	C9—C8—H8	109.5
C3—C2—H2	120.9	C10—C8—H8	109.5
C1—C2—H2	120.9	C8—C9—H9A	109.5
C2—C3—C4	123.15 (14)	C8—C9—H9B	109.5
C2—C3—N1	118.50 (13)	H9A—C9—H9B	109.5
C4—C3—N1	118.34 (14)	C8—C9—H9C	109.5
C3—C4—C5	117.92 (14)	H9A—C9—H9C	109.5
C3—C4—H4	121.0	H9B—C9—H9C	109.5
C5—C4—H4	121.0	C8—C10—H10A	109.5
C4—C5—C6	120.24 (13)	C8—C10—H10B	109.5
C4—C5—H5	119.9	H10A—C10—H10B	109.5
C6—C5—H5	119.9	C8—C10—H10C	109.5
C5—C6—C1	120.35 (13)	H10A—C10—H10C	109.5
C5—C6—C7	122.02 (13)	H10B—C10—H10C	109.5
C1—C6—C7	117.62 (14)		
			
C6—C1—C2—C3	−1.0 (2)	C4—C5—C6—C7	179.97 (14)
C1—C2—C3—C4	0.1 (2)	C2—C1—C6—C5	1.2 (2)
C1—C2—C3—N1	178.46 (13)	C2—C1—C6—C7	−179.10 (14)
O4—N1—C3—C2	177.20 (16)	C8—O1—C7—O2	2.8 (2)
O3—N1—C3—C2	−3.3 (2)	C8—O1—C7—C6	−176.78 (12)
O4—N1—C3—C4	−4.3 (2)	C5—C6—C7—O2	168.27 (16)
O3—N1—C3—C4	175.17 (15)	C1—C6—C7—O2	−11.5 (2)
C2—C3—C4—C5	0.8 (2)	C5—C6—C7—O1	−12.1 (2)
N1—C3—C4—C5	−177.64 (14)	C1—C6—C7—O1	168.17 (13)
C3—C4—C5—C6	−0.6 (2)	C7—O1—C8—C9	−96.71 (16)
C4—C5—C6—C1	−0.3 (2)	C7—O1—C8—C10	140.63 (15)

### Hydrogen-bond geometry (Å, °)

**Table:**

*D*—H···*A*	*D*—H	H···*A*	*D*···*A*	*D*—H···*A*
C1—H1···O4^i^	0.95	2.46	3.311 (3)	149
C4—H4···O2^ii^	0.95	2.46	3.294 (3)	147

Symmetry codes: (i) *x*, *y*−1, *z*; (ii) *x*, *y*+1, *z*.
